# CVD Diamond Interaction with Fe at Elevated Temperatures

**DOI:** 10.3390/ma11122505

**Published:** 2018-12-10

**Authors:** Sergei Zenkin, Aleksandr Gaydaychuk, Vitaly Okhotnikov, Stepan Linnik

**Affiliations:** Research School of High-Energy Physics, Tomsk Polytechnic University, Savinyh str. 2a, Tomsk 634050, Russia; gaydaychuk@tpu.ru (A.G.); ohotnikov@tpu.ru (V.O.); linniksa@tpu.ru (S.L.)

**Keywords:** CVD diamond, diffusion, Fe–C interaction

## Abstract

Chemical vapor deposition (CVD) diamond is a prospective thin film material for cutting tools applications due to the extreme combination of hardness, chemical inertness, and thermal conductivity. However, the CVD diamond cutting ability of ferrous materials is strongly limited due to its extreme affinity to iron, cobalt, or nickel. The diamond–iron interaction and the diffusion behavior in this system are not well studied and are believed to be similar to the graphite–iron mechanism. In this article, we focus on the medium-temperature working range of 400–800 °C of a CVD diamond–Fe system and show that for these temperatures etching of diamond by Fe is not as strong as is generally accepted. The starting point of the diamond graphitization in contact with iron was found around 400 °C. Our results show that CVD diamond is applicable for the cutting of ferrous materials under medium-temperature conditions.

## 1. Introduction

Chemical vapor deposition (CVD) diamond is a prospective thin film material for cutting tools applications due to the extreme combination of hardness, chemical inertness, and thermal conductivity [1]. However, the CVD diamond cutting ability of ferrous materials is strongly limited due to its extreme affinity to iron, cobalt, or nickel [2] attributed to the phase transformation of diamond to graphite and subsequent diffusion of carbon into the metal [3]. This phenomenon is frequently used for diamond catalytic etching [4,5], patterning [6], or polishing [7]. Jin et al. [8] report the thinning of CVD diamond, caused by the reaction with iron foil with a speed up to 2 µm/h at 900 °C in argon. Ralchenko et al. [9] show that for the CVD diamond–Fe system in a hydrogen atmosphere the etching speed increases up to 8 µm/min due to the formation of gaseous hydrocarbons, primarily methane. Giménez et al. [10] show an extreme increase of the chemical wear rate of polycrystalline diamond during iron-based materials machining in the temperature range from 700 to 1300 °C. Most of these experiments were carried out at extreme temperatures up to 1000 °C, while the diamond–iron interaction and diffusion behavior in the diamond–Fe system at lower temperatures are not well studied and are believed to be similar to the graphite–iron mechanism.

In the article, we are focused on the interaction between CVD grown microcrystalline diamond and thermally evaporated Fe at elevated temperatures in the range of 400–800 °C under vacuum conditions. We show that in this temperature range, etching of CVD diamond is not as strong as for the 900–1000 °C interval [8,10], making a CVD diamond applicable for the cutting of ferrous materials under medium-temperature conditions.

## 2. Materials and Methods

**Figure 1:**
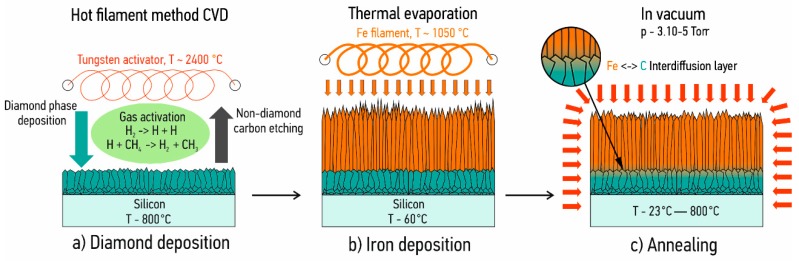
A schematic illustration of the experimental procedure: (**a**) Diamond deposition; (**b**) Iron deposition; (**c**) Annealing.

As a substrate in this work, we used monocrystalline Si (100) with the size of 10 mm × 10 mm × 0.38 mm. The diamond–iron system preparation is schematically shown in Figure 1. The samples were ultrasonically cleaned with acetone prior to diamond layer deposition.

Polycrystalline diamond coatings were deposited in the self-made hot filament chemical vapor deposition reactor using a hydrogen–methane mixture (ratio of H_2_:CH_4_ = 50:1) with a total gas flow rate 106 mL/min (Bronkhorst EL-FLOW, Bronkhorst High-Tech B.V., Ruurlo, The Netherlands). The distance between tungsten filaments (Ø = 0.16 mm) and substrates was 10 ± 0.5 mm. The substrate temperature during the deposition was maintained at 800 ± 25 °C using an infrared thermal imager (ULIRvision TI170, ULIRvision Technology Co., Ltd., Zhejiang, China). The pressure in the reactor during the deposition was maintained at 20 ± 1 Torr (Pfeiffer Vacuum CMR 372, Pfeiffer Vacuum, Annecy, France) and the current at 6.5 ± 0.01 A per filament, see Figure 1a. The total diamond film thickness was 2 ± 0.1 µm. 

After diamond layer synthesis, samples were placed in the vacuum chamber. The base pressure in the evacuated chamber was 8 × 10^−6^ Torr. Prior to Fe deposition, samples were cleaned by an Ar^+^ ion source with an energy of 3.5 keV.

Fe films were deposited by the two-step thermal evaporation process, see Figure 1b:

- An initial 3 µm layer was evaporated with ion source assistance using an Ar^+^ ion energy of 500 eV.
- Subsequently, Fe deposition was carried out without ion source assistance. The total Fe film thickness was 10 ± 0.5 µm.

After that, samples were annealed under vacuum conditions during 30 min at fixed temperatures in the range of 400–800 °C, see Figure 1c. In order to determine the interaction behavior between CVD diamond and evaporated Fe films, we used cross-sectional scanning electron microscopy, XRD, Raman spectroscopy, and EDX measurements. This combination of techniques allows for clarification of the interdiffusion process in the CVD diamond–Fe system at elevated temperatures and for the determination of the temperature starting point of the diamond graphitization. The coatings cross-sectional morphology was studied using a scanning electron microscope (Vega3, TESCAN, Brno, Czech Republic). Structural characteristics of the coatings were studied using X-ray diffraction (Shimadzu XRD 6000, Shimadzu, Kyoto, Japan) in the Bragg–Brentano configuration with Cu Kα (λ = 0.154 nm) radiation in the range of 2θ = 20°–90°, with a sampling pitch equal to 0.01°, and an integration time of 1 s. Raman spectra were recorded using a NanoScan Technology Centaur IHR spectrometer (NanoScan Technology, Dolgoprudny, Russia) with a 514.5 nm source laser.

## 3. Results and discussion

**Figure 2:**
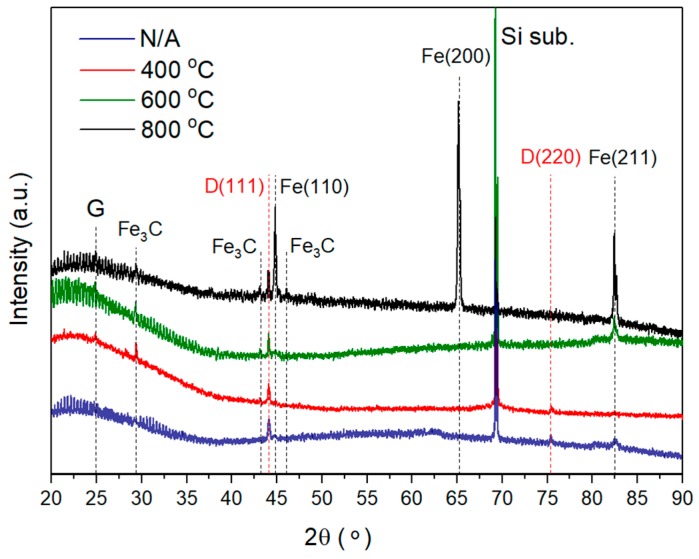
XRD patterns of the CVD diamond–Fe system at elevated temperatures.

The results of XRD measurements are shown in Figure 2. The CVD diamond–Fe system that was not annealed shows only diamond reflexes at 2θ = 44.05° and 75.4° attributed to the (111) and (220) crystallographic planes, respectively, in combination with low-intensity broad peaks of α-Fe at 2θ = 44.76° (Fe (110)) and 82.43° (Fe (211)). A strong change in the microstructure of the system can be created only after annealing above 400 °C, as shown in Figure 2. For this sample, one can detect the formation of the graphite phase with the peak position at 2θ ≈ 24.8° (“G” in Figure 2), indicating the start of the carbon diffusion into the iron layer. For a higher-temperature interval, 600–800 °C, we can detect the recrystallization of the thermally evaporated Fe film, starting at 600 °C, attributed to the formation of strong α-Fe peaks at 2θ = 44.76° (Fe (110)), 65.13° (Fe (200)) and 82.43° (Fe (211)). Analysis of the full width at half maximum (FWHM) of the α-Fe peak at 2θ = 82.43° using the Scherrer equation shows the coherent scattering region broadening from 23 to 38 nm, pointing to the increase of the crystallite size in the iron film. However, due to the limited ability of the Fe_3_C formation (only 25 at.% of carbon was involved in the formation of Fe_3_C), a strong peak due to α-Fe overlap appears at 2θ ≈ 45° in the XRD pattern. Only low-intensity Fe_3_C peaks at 2θ = 43.11° and 46.01° can be detected for a diamond–Fe system heated at 800 °C. At the same time, neither the intensity or the width of the diamond peak at 2θ = 44.05° changes as a result of heating the system, even at 800 °C. Based on the data, one can assume that only a minor amount of carbon from the CVD diamond film was involved in the formation of Fe_3_C or graphite during the interdiffusion process.

**Figure 3:**
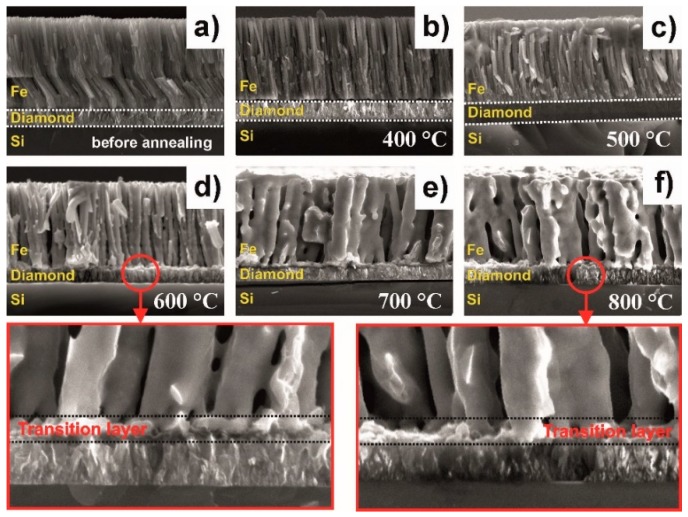
Cross-sectional scanning electron microscopy image of a CVD diamond–Fe system (**a**) as deposited, heated at (**b**) 400 °C; (**c**) 500 °C; (**d**) 600 °C; (**e**) 700 °C; and (**f**) 800 °C. The transition layer in the CVD diamond–Fe interface annealed at 600 °C (bottom left) and 800 °C (bottom right) during 30 min under vacuum conditions.

In order to support our assumption, we used scanning electron microscopy measurements of the cross-section of the CVD diamond–Fe interface as a function of the annealing temperature, see Figure 3. The iron films that were not annealed are characterized by a strongly columnar void-free microstructure and exhibit homogeneous interface contact with the diamond film, as shown in Figure 3a. For diamond–Fe systems heated in the range 400–500 °C, see Figure 3b,c, there is no visible change in the system structure and the formation of an Fe–C transition layer is not clearly observed, despite the fact that the formation of both graphite and Fe_3_C is well-evidenced in the XRD spectrum at 400 °C, see the red curve of Figure 2. Further increasing the annealing temperature, see Figure 2d–f, leads to:

- Recrystallization of the thermally evaporated Fe film, starting at 600 °C, in agreement with XRD measurements.
- Formation of an Fe–C transition layer due to the interdiffusion that takes place in the diamond–Fe system in the temperature range 600–800 °C. The thickness of this transition layer is relatively low (approx. 0.5 µm) due to the limited ability of Fe_3_C to form. Diffusion of carbon is undetectable on the SEM cross-sections.

**Figure 4:**
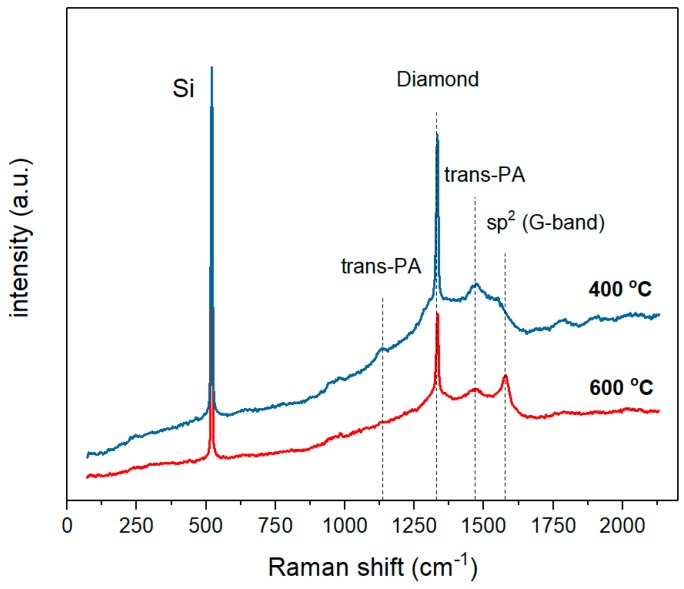
Raman spectra of the CVD diamond–Fe interface for films heated at 400 °C (blue) and 600 °C (red) indicating the formation of a graphite phase due to the carbon–iron interdiffusion.

To clarify the Fe–C interdiffusion, we measured the Raman spectra of the CVD diamond–Fe interface, highlighted in Figure 3 (marked as transition layer). To carry out the measurements, we chemically etched the top Fe layer using concentrated nitric acid. After that, we measured the Raman spectra from the top of the diamond film. The results of these measurements are shown in Figure 4. For samples annealed at 400 °C (blue curve) and 600 °C (red curve), one can detect three strong peaks at 1333 cm^−1^, 1471 cm^−1^, and 1578 cm^−1^, attributed to the diamond (pure *sp*^3^–hybridization of carbon), trans-polyacetylene, and G–band (graphitic carbon structure), respectively. It can be seen that, as the annealing temperature increases, the Raman spectra of 600 °C-heated samples clearly exhibit a decrease of the diamond peak intensity at 1333 cm^−1^ and an increase of the non–diamond peak centered at 1578 cm^−1^.

**Figure 5:**
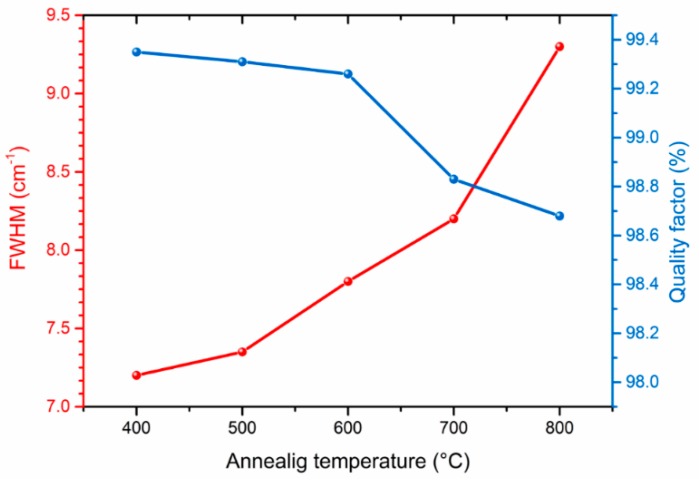
FWHM of the Raman diamond peak at 1333 cm^−1^ and the corresponding diamond quality factor as a function of the annealing temperature.

In order to make a quantitative evaluation of the changes in the quality of the diamond film, we investigated a change of the diamond Raman peak width. The obtained data are presented in Figure 5.

As can be seen, increasing the annealing temperature of the diamond–iron system from 400 to 800 °C does not lead to a significant deterioration in the quality of the diamond coating. The diamond peak FWHM for 400 °C is 7.2 cm^−1^, while for 800 °C, the annealed film is around 9.3 cm^−1^ (FWHM = 2 cm^−1^ for the highest available quality type IIa diamond [11]). The diamond phase purity was also quantified by the calculation of the diamond quality factor described as:

(1) $$Q=\frac{I_{\mathrm{diamond}}}{\left(I_{\mathrm{diamond}}+\frac{I_{\mathrm{a-carbon}}}{233}\right)}\times 100\%$$

here *I*_diamond_ is the diamond peak intensity at 1333 cm^−1^, *I*_a-carbon_ is the sum of the intensities of the observed nondiamond carbon lines [12].

As it is shown in Figure 5, the diamond quality factor is in the range of 98.7%–99.4% for the temperature interval 400–800 °C, confirming the high phase purity of the diamond film and a low interaction ability of the CVD diamond–Fe system in the investigated temperature range.

**Figure 6:**
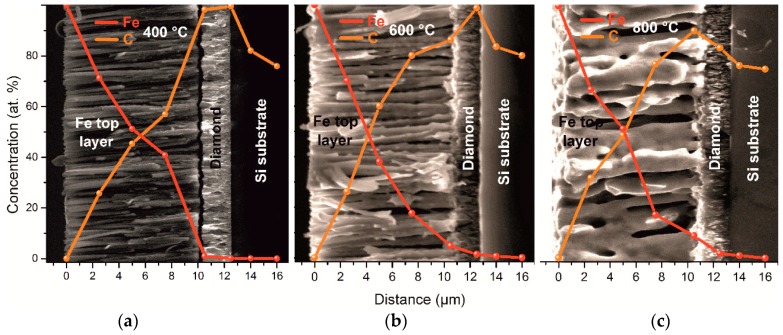
EDX plots of carbon and iron for the CVD diamond–Fe system annealed at (**a**) 400 °C; (**b**) 600 °C and (**c**) 800 °C.

**Figure 7:**
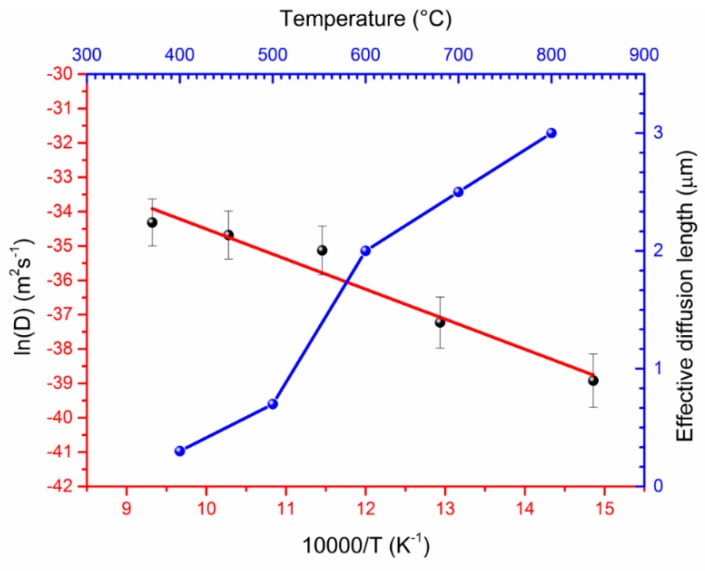
Arrhenius plot for Fe diffusion into the diamond layer and the corresponding diffusion length of the Fe as a function of the annealing temperature.

For a deeper understanding of the element distribution during the interdiffusion process, we measured concentrations of carbon and iron using the EDX method on the cross-section of the sintered diamond–Fe system. The results of these measurements are shown in Figure 6. We select three representative samples annealed at 400, 600, and 800 °C. The strong diffusion of carbon into the Fe volume starts even at temperatures lower than 400 °C due to its extreme diffusion coefficient [13], Figure 6a 25% of carbon was involved in the Fe_3_C formation while its residual part diffuses into the Fe bulk with the formation of graphite, in agreement with the XRD measurements shown in Figure 2. One can conclude, that at temperatures above 400 °C only the diffusion of carbon into the iron occurs. However, at higher temperatures (from 600 °C) diffusion of Fe into the diamond layer becomes substantial, see Figure 6b,c. The calculated effective diffusion length (*x*) of the Fe into the diamond film is represented in Figure 7. It was obtained from the distance between the top of the diamond layer and the point where the Fe concentration plot reaches 0%. EDS measurements were performed at five different points for each distance. The statistical deviation of the concentration measurement was ± 5 at.%. Due to the fact that we cannot detect a major change of the diamond layer using SEM measurements while Raman spectroscopy measurements show a minor reduction of the diamond quality parameter, we assume that diffusion occurs along the grain boundaries of the diamond layer. For the determination of the diffusion coefficient and diffusion activation energy of iron, we used Fick’s second law:

(2) $$D=\frac{x^{2}}{4t}$$

here *x* is the effective diffusion length of the Fe, *D* is the diffusion coefficient, and *t* is the diffusion time.

Diffusion coefficients of the Fe at different temperatures, Equation (2), were used to prepare the Arrhenius plot, as shown in Figure 7. From the intersection of the linear fits with the ordinate, we obtained the pre-exponential factor, *D*_0_, and derived the activation energy, *Q*, from their slope using the Arrhenius equation for the diffusion coefficient, *D*:

(3) $$D=D_{0}\exp\left(-\frac{Q}{RT}\right)$$

Here *D* is the Fe diffusion coefficient into the diamond film, *D*_0_ is the pre-exponential factor, *Q* is the diffusion activation energy, *R* is the gas constant, and *T* is the absolute temperature.

It can be seen that the diffusion coefficient of the Fe increases upon increasing the annealing temperature and changes from the 1.25 × 10^−17^ m^2^ s^−1^ at 400 °C up to 1.25 × 10^−15^ m^2^ s^−1^ at 800 °C. The pre-exponential factor and the activation energy obtained from the graph were 5.6 × 10^−12^ m^2^ s^−1^ and 69.1 kJ/mol, respectively.

## 4. Conclusions

To summarize, we can conclude that:

- The CVD diamond–Fe interaction in the range of 400–800 °C is significantly lower in comparison with previously reported data for the range of temperatures 900–1300 °C. Diamond etching and graphitization in the range of 400–800 °C are undetectable during microscopic measurements and can be detected only by XRD and Raman measurements.
- Annealing of the diamond in contact with Fe slightly reduces the quality parameter in the range of 98.7–99.4%, confirming a slight interaction of diamond–Fe at the selected temperature range.
- Strong diffusion of carbon into the Fe occurs even at low-temperature annealing conditions of 400 °C.
- Formation of an Fe–C transition layer due to the interdiffusion process in the diamond–Fe system can be detected in the temperature range 600–800 °C. The thickness of this transition layer is relatively low (approx. 0.5 µm) due to the limited ability of the Fe_3_C to form.
- When annealing at 600 °C, one can detect a diffusion of the Fe into the diamond film. The Fe diffusion coefficient was estimated from 1.25 × 10^−17^ m^2^ s^−1^ at 400 °C up to 1.25 × 10^−15^ m^2^ s^−1^ at 800 °C. The diffusion activation energy of the Fe was determined to be 69.1 kJ/mol.
